# Supplementary material for: Content-rich biological network constructed by mining PubMed abstracts
Source: BMC Bioinformatics. 2004 Oct 8;5:147. doi: 10.1186/1471-2105-5-147 (PMC528731; doi:10.1186/1471-2105-5-147)
Supplement: Additional File 2 — The original results of the above study (non-essential files are deleted to keep the file size under the limit set by BMC bioinformatics). [file 1471-2105-5-147-S2.bz2 › chilibotAdditionalFile2/dip05/312ID11278571E1364/html/ZIPA_FTSZ.html]

 


 **ZIPA** and **FTSZ** 
  
Found 24 abstracts in PubMed, retrieved 05.  
 

 What does Google say? 
 PDF only 
| .edu only 

---

**Interactive relationship** (e.g. stimulation, inhibition, etc)

**Neutral relationship**- The septal ring in Escherichia coli consists of at least nine essential gene products whose order of assembly resembles a mostly linear dependency pathway FtsA and  **ZipA**  directly bind  **FtsZ**  polymers at the prospective division site, followed by the sequential addition of FtsK, FtsQ, FtsL, FtsW, FtsI, and FtsN.  Ref: 11948172 J Bacteriol, 2002
- FtsA and  **ZipA**  localized into a division ring following the same pattern and time course as  **FtsZ** .  Ref: 12754232 J Bacteriol, 2003
- The cell division protein  **ZipA**   [ **ZIPA** ]  has an N terminal transmembrane domain and a C terminal globular domain that binds  **FtsZ** .  Ref: 12107152 J Bacteriol, 2002
- Structural evidence that the P Q domain of  **ZipA**  is an unstructured, flexible tether between the membrane and the C terminal  **FtsZ**  binding domain.  Ref: 12107152 J Bacteriol, 2002

**Non-interactive relationship** (e.g. studied together, co-existance, homology, etc.)

- Concentration and Assembly of the Division Ring Proteins  **FtsZ** , FtsA, and  **ZipA**  during the Escherichia coli Cell Cycle.  Ref: 12754232 J Bacteriol, 2003
- **ZipA**  and FtsA are recruited independently to the  **FtsZ**  cytokinetic ring Z ring and are essential for cell division of Escherichia coli.  Ref: 12634424 Proc Natl Acad Sci U S A, 2003
- The concentration of the cell division proteins  **FtsZ** , FtsA, and  **ZipA**  and their assembly into a division ring during the Escherichia coli B r K cell cycle have been measured in synchronous cultures obtained by the membrane elution technique.  Ref: 12754232 J Bacteriol, 2003
- The results imply that association of both FtsA and  **ZipA**  with  **FtsZ**  polymers is needed for further maturation of the nascent organelle.  Ref: 11948172 J Bacteriol, 2002
